# Influence of Dietary Supplementation with an Amino Acid Mixture on Inflammatory Markers, Immune Status and Serum Proteome in LPS-Challenged Weaned Piglets

**DOI:** 10.3390/ani11041143

**Published:** 2021-04-16

**Authors:** José A. M. Prates, João P. B. Freire, André M. de Almeida, Cátia Martins, David M. Ribeiro, Hugo Osório, Mário A. S. Pinho, Paula A. Lopes, Jorge M. J. Correia, Rui M. A. Pinto, Teresa Costa, Etienne Corrent, Tristan Chalvon-Demersay

**Affiliations:** 1 CIISA—Centro de Investigação Interdisciplinar em Sanidade Animal, Faculdade de Medicina Veterinária, Universidade de Lisboa, Avenida da Universidade Técnica, Alto da Ajuda, 1300-477 Lisboa, Portugal; japrates@edu.ulisboa.pt (J.A.M.P.); catiamartins@isa.ulisboa.pt (C.M.); mpinho@fmv.ulisboa.pt (M.A.S.P.); ampalopes@fmv.ulisboa.pt (P.A.L.); jcorreia@fmv.ulisboa.pt (J.M.J.C.); 2 LEAF—Linking Engineering, Agriculture and Food, Departamento de Ciências e Engenharia de Biossistemas, Instituto Superior de Agronomia, Universidade de Lisboa, Tapada da Ajuda, 1349-017 Lisboa, Portugal; jpfreire@isa.utl.pt (J.P.B.F.); aalmeida@isa.ulisboa.pt (A.M.d.A.); davidribeiro@isa.ulisboa.pt (D.M.R.); 3 i3S—Instituto de Investigação e Inovação em Saúde, Universidade do Porto, 4200-135 Porto, Portugal; hosorio@i3s.up.pt; 4 Ipatimup—Institute of Molecular Pathology and Immunology of the University of Porto, University of Porto, 4200-135 Porto, Portugal; 5 Department of Pathology, Faculty of Medicine, University of Porto, 4200-319 Porto, Portugal; 6 iMed UL, Faculdade de Farmácia, Universidade de Lisboa, Av. Professor Gama Pinto, 1649-003 Lisboa, Portugal; rapinto@ff.ulisboa.pt; 7 JCS—Laboratório de Análises Clínicas Dr. Joaquim Chaves, Av. General Norton de Matos, 1495-148 Miraflores, Portugal; 8 Indukern Portugal, Lda., Centro Empresarial Sintra Estoril II, Rua Pé de Mouro, Edifício C, Apartado 53, Estrada de Albarraque, 2710-335 Sintra, Portugal; teresa.costa@indukern.pt; 9 Ajinomoto Animal Nutrition Europe, 32 rue Guersant, 75017 Paris, France; corrent_etienne@eli.ajinomoto.com; 10 Ajinomoto Co., Inc., Tokyo 104-8315, Japan

**Keywords:** piglets, amino acids, immunity, challenge

## Abstract

**Simple Summary:**

Piglets at weaning face numerous changes: they are separated from the sow, are transitioned from milk to solid feed, and are frequently mixed with other litters in a totally new environment. In addition, they are predisposed to bacterial infections. In this context, there is a strong need to develop mitigating dietary strategies to limit the impact of these changes, which alter performance and immune status. In our study, we mimicked a mild bacterial infection in weaned piglets by intraperitoneally administering lipopolysaccharide (LPS), a bacterial endotoxin. Half of the challenged piglets were fed with a diet supplemented with a mixture of functional amino acids. Our study showed that LPS challenge increased markers of inflammation in piglets, which could be partially reversed by the supplementation of the amino acid mixture supplementation. These data indicate that the amino acid mixture supplementation could have a protective effect for challenged piglets during weaning.

**Abstract:**

In order to investigate the effect of a dietary amino acid mixture supplementation in lipopolysaccharide (LPS)-challenged weaned piglets, twenty-seven 28-day-old (8.2 ± 1.0 kg) newly weaned piglets were randomly allocated to one of three experimental treatments for five weeks. Diet 1: a CTRL treatment. Diet 2: an LPS treatment, where piglets were intraperitoneally administered LPS (25 μg/kg) on day 7. Diet 3: an LPS+MIX treatment, where piglets were intraperitoneally administered LPS on day 7 and fed a diet supplemented with a mixture of 0.3% of arginine, branched-chain amino acids (leucine, valine, and isoleucine), and cystine (MIX). Blood samples were drawn on day 10 and day 35, and serum was analysed for selected chemical parameters and proteomics. The LPS and LPS+MIX groups exhibited an increase in haptoglobin concentrations on day 10. The LPS group showed an increased cortisol concentration, while this concentration was reduced in the LPS+MIX group compared to the control group. Similarly, the LPS+MIX group showed a decreased haptoglobin concentration on day 35 compared to the two other groups. Immunoglobulin concentrations were affected by treatments. Indeed, on day 10, the concentrations of IgG and IgM were decreased by the LPS challenge, as illustrated by the lower concentrations of these two immunoglobulins in the LPS group compared to the control group. In addition, the supplementation with the amino acid mixture in the LPS+MIX further decreased IgG and increased IgM concentrations compared to the LPS group. Although a proteomics approach did not reveal important alterations in the protein profile in response to treatments, LPS-challenged piglets had an increase in proteins linked to the immune response, when compared to piglets supplemented with the amino acid mixture. Overall, data indicate that LPS-challenged piglets supplemented with this amino acid mixture are more protected against the detrimental effects of LPS.

## 1. Introduction

Weaning is a critical period in which piglets face many important challenges that will determine their subsequent health and productivity. In fact, once they are removed from the sow, they are transitioned from milk to solid feed and are frequently mixed with other litters in a totally new environment [1], thus being exposed to different microbial and sanitary environments. These changes generate the so-called weaning stress that leads to a dramatic reduction in water and feed intake [2,3], potentially compromising the animal’s future productivity. Indeed, it has been reported that 50% of piglets do not consume any feed in the first 24 h post-weaning [4]. As a consequence, signs of dysbiosis can be noticed and are characterised by the proliferation of opportunistic pathogens such as *Escherichia coli* [5]. In addition, the interaction of lipopolysaccharides (LPS), located in the outer membrane of *E. Coli*, with the transmembrane receptor toll-like receptor 4 (TLR-4), can trigger inflammation [6]. These changes lead to animals’ discomfort, diarrhoea, growth stunting, and increased mortality.

In addition to being the building blocks of protein synthesis, amino acids are key functional and signalling molecules in the body and are involved in the regulation of oxidative stress, immunity, and intestinal barrier function [7]. Evidence of the roles of amino acids to mitigate the consequences of weaning has already been described in the literature. Thus, dietary arginine supplementation in piglets facing an LPS challenge leads to an increase in villus height in jejunum and ileum [8]. Moreover, it regulates oxidative stress status by increasing ferric reducing ability in plasma and decreasing the oxidised form of glutathione [9]. Under the same experimental model of challenge, cysteine supplementation has been associated with an increase in transepithelial resistance and a decrease in pro-inflammatory cytokine secretion [10]. Finally, dietary supplementation with branched-chain amino acids (BCAAs) is associated with an improvement of gut morphology in piglets at weaning, as well as with an increase in the expression of amino acid transporters [11,12]. Taken together, these results suggest that these amino acids have complementary modes of action and could help piglets coping with weaning and LPS challenge.

However, to the best of our knowledge, no study has specifically investigated the specific dietary supplementation of these amino acids, when supplemented as a mixture, to recently weaned piglets facing an inflammatory-immunological challenge. The objective of this work was, therefore, to assess the effect of a specific combination of these amino acids on mitigating a mild LPS challenge in recently weaned piglets, through the determination of inflammatory markers, immune status, and serum proteome.

## 2. Materials and Methods

### 2.1. Animal Welfare Disclaimer

The experimental procedures were reviewed and approved by the Animal Care Committee of ISA—Instituto Superior de Agronomia, Universidade de Lisboa, (Lisbon, Portugal) and authorised by the National Veterinary Authority (Direcção Geral de Alimentação e Veterinária (Lisbon, Portugal), following the appropriate European Union guidelines (2010/63/EU Directive). Ethical approval code is #0421/000/000/2017. J.A.M. Prates, A.M. Almeida, P.A. Lopes and J.M.J. Correia each hold a FELASA grade C certificate, which enables them to design and conduct animal experimentation in the European Union.

### 2.2. Animals, Diets and Sampling

This trial was performed at the Animal Production Sector of, ISA, Universidade de Lisboa (Lisbon, Portugal). Twenty-seven 28-day-old newly weaned male crossbred piglets (F2 crosses of Pietrain × F1 [Landrace × Large white] crosses) with an initial body weight of 8.2 ± 1.0 kg (mean ± SD), were randomly assigned, on day 0, to different experimental diets. Piglets were submitted to one of three groups (CTRL, LPS and LPS+MIX) during a five-week period. A basal diet was formulated to contain 20% crude protein level and a level of 1.35% standardised ileal digestible Lys. The levels of standardised ileal digestible methionine, threonine, tryptophan, valine, isoleucine, leucine, histidine and phenylalanine+tyrosine relative to lysine followed those of NRC 2012 [13] recommendations. For the LPS+MIX group, this basal diet was supplemented on-top with an 0.3% as-fed-basis of an AA mixture containing arginine, BCAA, and cystine, in a weight ratio of 42:33:25, as reported in Table 1. The mixture composition was derived from an initial mixture composition where arginine, BCAA, and cystine contribution was equal. Cystine contribution was then decreased at the expense of arginine because cystine is known to have mucolytic effects above a certain dose [2], while arginine is very well tolerated at high doses. After 7 days, all piglets, except the ones from the CTRL group, which were injected with a sterile saline solution, received a mild LPS challenge administered intraperitoneally at a dosage of 25 μg/kg of body weight (25 µL/kg of body weight) [14]. The LPS reagent, extracted and purified from *Escherichia coli* O55:B5, was acquired from Sigma-Aldrich (St. Louis, MO, USA). During the experimental period, piglets were housed in pens with three animals, fed ad libitum with pelleted feed, and weighed individually once a week. Diarrhoea was monitored at the pen level by following the mean consistency of faeces, as described by Marquardt et al. [15]: normal = 0, soft diarrhoea = 1, mild diarrhoea = 2, and severe diarrhoea = 3. To investigate the medium-term effects of LPS injection, three days after the challenge (on day 10) and at the end of the experimental period (on day 35), blood samples were drawn from the jugular veins of piglets onto Z Gel 4.5 mL Sarstedt (Nümbrecht, Germany) test tubes after a 4 h fast. Blood was centrifuged (1500× *g* for 10 min at room temperature) to harvest serum. The serum was snap-frozen in liquid nitrogen and stored at −80 °C until further analysis. At the end of the experimental period, on day 35, all piglets were sacrificed, and intestinal mucosa were sampled as described thereafter.

### 2.3. Gut Morphology

Gut morphology tissue samples were collected from the duodenum (10 cm from the pylorus), the jejunum (5.5 m from the pylorus), and the ileum (60 cm before the ileo-caecal valve), and processed as described elsewhere [16]. Briefly, the tissue samples were fixed in 10% neutral buffered formalin and embedded in paraffin wax for microscopic examination of the intestinal villi and crypts. A BX 511 microscope (Olympus, Tokyo, Japan) was used, and the images were digitally captured using a DP 11 camera (Olympus, Tokyo, Japan) under a magnification of 40×. The height and width of the villi and the depth of the crypts were measured using the DP-Soft software (Olympus). Ten intact and properly oriented villi and crypts from each intestinal region were selected per piglet.

### 2.4. Serum Immune and Hormonal Status

Tumour necrosis factor-alpha (TNF-α) was measured by ELISA commercial kit (R&D Systems, Minneapolis, MN, USA). This kit contains *E. coli*-expressed recombinant porcine TNF-α and antibodies raised against the recombinant factor. Results obtained using natural porcine TNF-α show dose–response curves that are parallel to the standard curves obtained using the kit standards. Insulin growth factor-1 (IGF1) was determined by a chemiluminescent immunoassay kit (ImmunoDiagnostic Systems, Paris, France).

The immunoglobulins profile, including IgA, IgG and IgM, was determined by immunoturbidimetry (Biosystems, Barcelona, Spain). The light scattering of antigen–antibody complexes is proportional to the immunoglobulins concentration and can be measured by turbidimetry.

Haptoglobin was quantified by immunoturbidimetry (Kamiya Biomedical Company, Seattle, WA, USA). The antiserum used in the kit was produced against purified haptoglobin. The haptoglobin antibody interacts with the haptoglobin in the serum, forming immune complexes and causing an increase in light scattering which correlates with the concentration of serum haptoglobin. IGF1 was determined using a chemiluminescent immunoassay kit. The microplate provided in this kit had been pre-coated with an antibody specific to IGF1. Standards or samples were then added to the appropriate microplate wells with a biotin-conjugated antibody specific to IGF1. Next, avidin conjugated to horseradish peroxidase (HRP) was added to each microplate well and incubated. Then, the mixture of substrates was added to generate glow light emission kinetics. Upon plate development, the intensity of the emitted light was proportional to the IGF1 level in the samples.

Cortisol was quantified by electrochemiluminescence immunoassay method (Roche Diagnostics, Mannheim, Germany). The cortisol assay is a competitive electrochemiluminescence immunoassay that uses a sheep polyclonal antibody. Endogenous cortisol contained in the sample is liberated from the binding proteins by danazol and, subsequently, competes with a cortisol derivative (a cortisol–peptide–Tris bipyridyl ruthenium complex) for the binding sites on the biotinylated antibody. After the addition of streptavidin-coated paramagnetic particles, the biotin on the antibody can bind to the streptavidin of the microparticle and form a complex. This complex is then captured on the surface of the magnetic electrode. Electrical stimulation of the ruthenium complex induces chemiluminescent emission, which is measured by a photomultiplier. The assay was calibrated against Enzymun-Test-Cortisol, which, in turn, was calibrated via isotope dilution mass spectrometry. The cortisol assay was used as instructed by the manufacturer without modifications.

### 2.5. Statistical Analysis for Non-Proteomics Data

All non-proteomics data were checked for normal distribution (Shapiro–Wilk test) and for variance homogeneity (Levene’s test). The piglet was the experimental unit for each variable, except for feed intake and faecal score, where the pen was used as the experimental unit. When data were normally distributed, they were analysed using one-factor ANOVA with treatment as the fixed factor followed by post hoc Tukey tests. If this was not the case, Kruskal–Wallis test was performed. Statistical analysis was performed using R software. The level of significance was set at *p* < 0.05, and trends were considered for *p* < 0.10.

### 2.6. Proteomics Analysis

Five serum samples from d35 were randomly selected from each of the three groups and used for proteomics analysis. The analysis was conducted as an external service from the Proteomics Scientific Platform of the i3S institute of the University of Porto (https://www.i3s.up.pt/scientific-platform?v=56, accessed on 14 September 2020, Porto, Portugal). Each sample was reduced and alkylated and processed for proteomics analysis following the solid-phase-enhanced sample-preparation (SP3) protocol, as described by Hughes et al. [17]. Enzymatic digestion was performed with Trypsin/LysC (2 µg) overnight at 37 °C, under constant shaking.

The protein identification and quantitation were performed by nano LC-MS/MS as previously described [18]. This equipment is composed of an Ultimate 3000 liquid chromatography system coupled to a Q-Exactive Hybrid Quadrupole-Orbitrap mass spectrometer (Thermo Scientific, Bremen, Germany). Samples were loaded onto a trapping cartridge (Acclaim PepMap C18 100A, 5 mm × 300 µm i.d., 160454, Thermo Scientific) in a mobile phase of 2% acetonitrile, 0.1% formic acid at 10 µL/min. After 3 min loading, the trap column was switched in-line to a 50 cm by 75 μm inner diameter EASY-Spray column (ES803, PepMap RSLC, C18, 2 μm, Thermo Scientific, Bremen, Germany) at 250 nL/min. The separation was generated by mixing A: 0.1% formic acid and B: 80% acetonitrile, with the following gradient: 5 min (2.5% B to 10% B), 120 min (10% B to 30% B), 20 min (30% B to 50% B), 5 min (50% B to 99% B) and 10 min (hold 99% B). Subsequently, the column was equilibrated with 2.5% B for 17 min. Data acquisition was controlled by Xcalibur 4.0 and Tune 2.9 software (Thermo Scientific).

The mass spectrometer was operated in data-dependent (dd) positive acquisition mode alternating between a full scan (*m/z* 380–1580) and subsequent higher-energy C-trap dissociation MS/MS of the 10 most intense peaks from full scan (normalised collision energy of 27%), as previously described [18]. ESI spray voltage was 1.9 kV. Global settings: use lock masses best (*m/z* 445.12003), lock mass injection Full MS, chromatography peak width (FWHM) 15 s. Full scan settings: 70k resolution (*m/z* 200), AGC target 3 x 10^6^ maximum injection time 120 ms. dd settings: minimum AGC target 8 × 10^3^, intensity threshold 7.3 x 10^4^, charge exclusion: unassigned, 1, 8, >8, peptide match preferred, exclude isotopes on, dynamic exclusion 45 s. MS2 settings: microscans 1, resolution 35k (*m/z* 200), AGC target 2 x 10^5^, maximum injection time 110 ms, isolation window 2.0 *m/z*, isolation offset 0.0 *m/z*, spectrum data type profile.

The raw data were processed using Proteome Discoverer 2.4.0.305 software (Thermo Scientific) and matched with the UniProt database for the *Sus scrofa* Proteome 2020_01. The Sequest HT search engine was used to identify tryptic peptides, as described by Matos et al. [18]. The ion mass tolerance was 10 ppm for precursor ions and 0.02 Da for fragment ions. The maximum allowed number of missing cleavage sites was set as 2. Cysteine carbamidomethylation was defined as constant modification. Methionine oxidation and protein N-terminus acetylation were defined as variable modifications. Peptide confidence was set to high. The processing node Percolator was enabled with the following settings: maximum delta Cn 0.05; decoy database search target FDR 1%, validation based on q-value. Protein label-free quantitation was performed with the Minora feature detector node at the processing step. Precursor ion quantification was performed at the processing step with the following parameters: peptides used unique plus razor, precursor abundance was based on intensity, normalisation mode was based on total peptide amount, protein ratios were directly calculated from the grouped protein abundances, and hypothesis test was based on ANOVA (individual proteins). Principal component analysis (PCA) results of the three experimental groups were plotted. Then, two comparisons were performed: CTRL vs. LPS and LPS vs. LPS+MIX. It was determined that proteins had differential abundance when *p* < 0.05 and a fold change of at least 1.5 was achieved [19].

### 2.7. Data Availability

All data generated during this study are included in this publication. The datasets generated during the current study are available in Table S1.

## 3. Results

### 3.1. Growth Performance and Faecal Score

The focus of this study was not performance, because the sample size was rather small for performance data collection. LPS or amino acid mixture supplementation (LPS+MIX) did not significantly affect (*p* > 0.05) faecal score (data not shown), body weight, average daily gain, or feed intake (Table 2).

### 3.2. Gut Morphology

**Figure 1:**
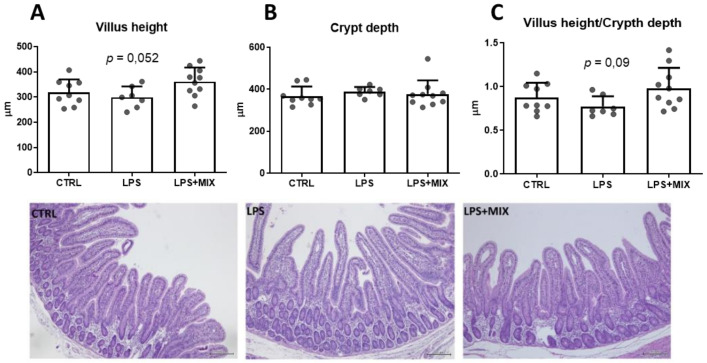
Gut morphology in jejunum at day 35, the end of the trial. (**A**–**C**) represent villus height, crypt depth and villus height/crypt depth ratio, respectively. Groups are: CTRL, the control group; LPS, the LPS-challenged group; LPS+MIX, the LPS-challenged supplemented with arginine, branched-chain amino acids (BCAA), and cystine mixture group (*n* = 7–9).

LPS challenge did not significantly affect gut morphology, either in the jejunum (Figure 1) or in duodenum plus ileum (data not shown). Conversely, the addition of amino acid mixture tended to increase villus height and the villus height/crypt depth ratio in the jejunum (*p* = 0.052 and *p* = 0.090, respectively) (Figure 1A,C). Representative pictures of jejunal sections from the three groups can be found in Figure 1.

### 3.3. Serum Inflammatory and Hormonal Status

The LPS group had an increased serum concentration of haptoglobin, an acute phase protein, on day 10 (*p* < 0.001) compared to the control group. Other inflammatory markers were not affected by the LPS challenge. Interestingly, the supplementation of the amino acid mixture in the LPS+MIX group reduced haptoglobin on day 35 (*p* = 0.022) but not on day 10 (*p* > 0.05) compared to the LPS group.

Regarding hormonal status, the LPS group showed an increase in cortisol (*p* < 0.001) and IGF-1 (*p* < 0.001) concentrations on day 10 (Table 3) compared to the control group. The dietary supplementation with the amino acid mixture decreased serum cortisol level (*p* < 0.001) and further increased IGF-1 concentration (*p* < 0.001), compared to the two other groups group. On day 35, cortisol concentration did not differ among groups (*p* > 0.05), but IGF-1 level was still increased in response to the dietary supplementation with the amino acid mixture (*p* < 0.001).

**Figure 2:**
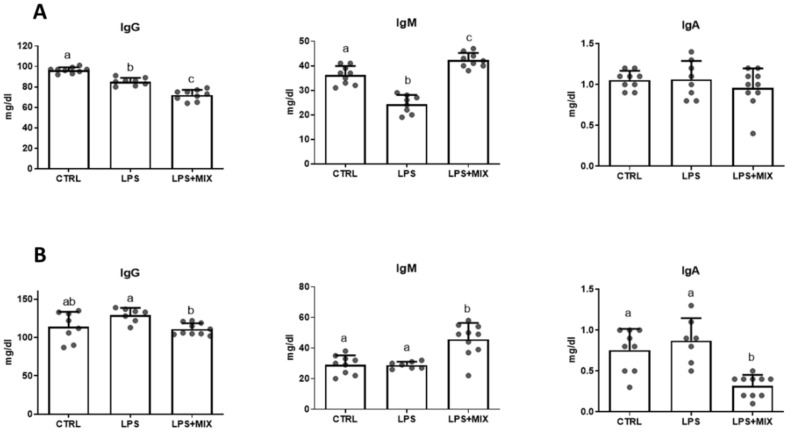
Serum concentrations of immunoglobulins IgG, IgM, and IgA on day 10 ((**A**)—upper panel) and day 35 ((**B**)—lower panel). Groups are: CTRL, the control group; LPS, the LPS-challenged group; LPS+MIX, the LPS-challenged supplemented with arginine, branched-chain amino acids (BCAA) and cystine mixture group. a,b,c: Histograms with different letters are significantly different (*p* < 0.05).

Immunoglobulin concentrations were affected by the experimental treatments (*p* < 0.05) (Figure 2). Indeed, on day 10, IgG and IgM concentrations were decreased (*p* < 0.05) in the LPS+MIX group compared to the control. The supplementation with the amino acid mixture in the LPS+MIX further decreased IgG and increased IgM concentrations compared to the LPS group.

On day 35, the immunoglobulins concentrations did not differ between the LPS and control groups. The dietary supplementation with the amino acid mixture increased IgM (*p* < 0.001) and decreased IgA (*p* < 0.001) concentrations, in comparison to the two other groups.

### 3.4. Serum Proteomics

**Figure 3:**
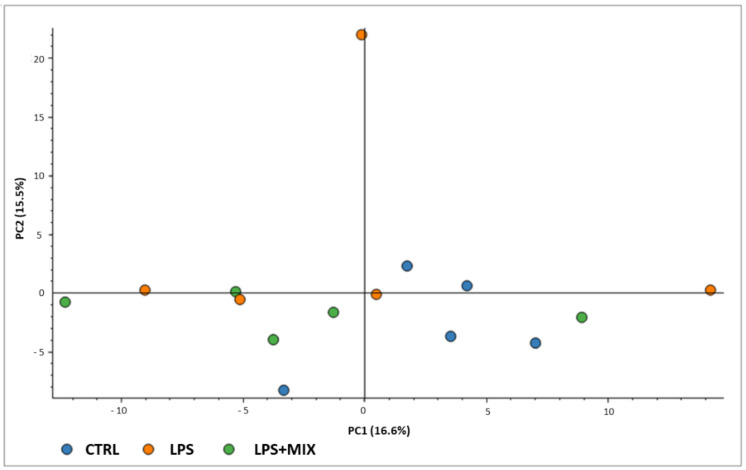
Principal component analysis (PCA) scatterplot of the three experimental groups. Groups are: CTRL, the control group; LPS, the LPS-challenged group; LPS+MIX, the LPS-challenged supplemented with arginine, branched-chain amino acids (BCAA) and cystine mixture group.

In total, over 200 different proteins were identified using the proteomics approach. The full proteomics results may be seen in Table S1. The proteomics results are presented as a PCA plot in Figure 3. Groups could not be clustered.

Differences are highlighted in Table 4. The comparison between LPS and CTRL groups revealed an accumulation of four proteins in the CTRL group. These proteins are inter-alpha-trypsin inhibitor heavy chain H2, von Willebrand factor, and two C1q domain-containing proteins. The comparison between LPS+MIX and LPS groups revealed seven proteins showing differential accumulation. Except for Complement component C8A, all proteins had a higher concentration in the LPS group.

## 4. Discussion

This study aimed to investigate whether dietary supplementation of a specific combination of arginine, BCAA, and cystine could be beneficial for piglets facing weaning under LPS challenge. LPS challenge was used in this study because it partially mimics the infection of *E. coli*, which is frequent in the post-weaning phase in piglets [5]. We made the choice to use a low dosage of LPS challenge (25 µg/kg), contrary to the majority of published studies, because we wanted to induce a mild challenge and inflammation without affecting mortality or generating severe diarrhoea [14]. In addition, the level of supplementation of the amino acid mixture in this trial was set to 0.3% as-fed-basis, so that this strategy could be easily implemented on commercial farms.

The results revealed that the dietary amino acid mixture supplementation was associated with a slight improvement of some gut morphology markers in jejunum, which could have contributed to a better nutrient absorption. The beneficial effects of arginine, BCAA, and cysteine supplemented solely on gut morphology in piglets has previously been described in the literature [8,9,10,11], and thus is in accordance with our results. However, the dietary supplementation of these amino acids in a mixture does not enable the identification of possible specific amino acids that are driving the response.

Our study indicates that LPS injection can modify inflammatory markers, as shown by the increase in serum cortisol and haptoglobin, which is in line with previous publications [20,21]. These quoted studies reported that intraperitoneal injection of LPS was also associated with a transient increase in TNF-α in serum, an observation which could not be seen in our study, which is likely due to the time of sampling. Interestingly, in our study, the addition of the amino acid mixture decreased cortisol to a concentration lower than that of the control animals. The combined effect of arginine and cystine could be responsible for this observation, as was previously described in piglets, in which the supplementation of these amino acids or their analogues lowered plasma cortisol levels [22,23,24].

Immunoglobulins (IgM, IgA, and IgG) are all produced by B lymphocytes but have specific structures and functions. IgM is a pentamer involved in the primary response against foreign substances, mediated by the activation of the complement system; IgA is a monomer serving as a gate keeper, driving mucosal response against pathogens; and IgG, also depicting a monomeric structure and the most abundant immunoglobulin, is involved in the second response against toxins and virus [25]. These immunoglobulins are known to mediate the immune response during an LPS challenge [26]. Our study showed that LPS injection led to a decrease in IgG and IgM in plasma three days after the challenge. Reduction in total IgM was previously reported following a challenge with *Escherichia coli* LPS in calves, but was accompanied with an increase in total IgG [26]. A similar shift of IgG towards IgM was observed in the amino acid supplemented group, although occurring earlier than reported by Kim and colleagues [26]. This could rely on a modification in lymphocyte subtypes [26]. In line with this study, the LPS challenge did not affect IgA concentration in plasma [26]. Interestingly, dietary amino acid supplementation reduced IgA concentration at the end of our study, when compared to the two other groups. Dietary arginine supplementation has been reported to increase IgA in the intestine mucosa and lumen [27]. We suggest that dietary arginine supplementation led to higher secretion of IgA in the lumen, which reduces its concentration in plasma. Unfortunately, we did not measure IgA concentration in the gut mucosa or lumen.

IGF-1 is a hormone with a molecular structure similar to insulin, which plays an important role in protein synthesis and exerts anabolic effects in the muscle via the regulation of the mTOR signalling pathway [28]. The effects of LPS challenge on IGF-1 secretion described in the literature are quite inconsistent; although an article reported an increase in IGF-1 in response to LPS challenge [29], as in our own study, others have reported the opposite results in rodents and pigs [30,31]. The increase in IGF-1 concentration in response to the dietary supplementation of the amino acid mixture could be due to the arginine stimulating effect on IGF-1 secretion, which is already well documented in the literature [32].

Surprisingly, while inflammatory-immunological analyses revealed differences among experimental groups, the proteomics approach did not reveal important alterations of protein profiles in response to the experimental treatment. Indeed, the PCA confirmed that treatments led to little changes in serum proteome of the piglets, because no clustering was visible that discriminated the three experimental groups. Still, some interesting results were recorded from the proteomics experiment. All the proteins showing differential accumulations in response to the LPS challenge are poorly described, except for the von Willebrand factor (vWF). This protein is, among other roles, involved in cell substrate adhesion [33], particularly platelets. In recent years, the role of the vWF in vascular inflammation and homeostasis has also been suggested [34]. As for the inter-alpha-trypsin inhibitor heavy chain H2 protein, this group of proteins has long been associated with the serine-type endopeptidase inhibitor activity and the hyaluronan (HA) metabolic process [35], both of which are highly relevant in tissue growth and regeneration [36]. Furthermore, according to Volpi et al. [37], HA is involved in several key processes, including cell signalling, morphogenesis, and wound repair and regeneration. Regarding the C1q domain-containing protein, it is also involved in the immune response [38]. All the above-mentioned proteins have decreased abundance in the LPS challenge group, when compared with the reference group. One can speculate that biological processes associated with cell substrate adhesion, HA metabolism, cell signalling, and growth were impaired by the LPS treatment. Such a pattern of results is indeed consistent with the LPS challenge to which the piglets were exposed. In turn, the comparison between LPS+MIX and LPS groups revealed several proteins showing differential accumulation. The first, uncharacterised protein OS=Sus scrofa, is a poorly studied protein with unknown molecular function (https://www.uniprot.org/uniprot/A0A287B5G4, accessed on 14 September 2020). The second protein, Complement C1q B chain, is part of a group of proteins involved in complement response regulation, specifically in the response to bacterial infection [39]. Similarly, Beta-2-microglobulin is involved in immune surveillance and modulation in vertebrates [40], specifically in antigen processing and the presentation of peptide antigens via major histocompatibility complex class I. As previously stated, uncharacterised protein OS=Sus scrofa OX=9823 is involved in the glutathione metabolic process and hydrogen peroxide reduction. Finally, Cathepsin B has long been demonstrated to play a major role in the regulation of proteolytic activity [41]. Interestingly, the results for Complement component C8A (A0SEH1) are not in accordance with the above-mentioned pattern of results. In fact, this is a protein involved in complement activation linked to different steps of the complement cascade and the regulation of immune processes [42,43]. The pattern of results shown in the LPS and LPS+MIX comparison indicates that piglets in the LPS group had an increased abundance of proteins linked to the immune response and to the regulation of immune processes. This could be because piglets in the LPS group probably established a more pronounced immune response, as a consequence of the microbial infection-induced stress mimicked by the LPS injection, when compared with piglets receiving the amino acid mixture. This, in turn, may indicate that the piglets supplemented with the mixture of amino acids were less susceptible to the negative effects of the LPS challenge in comparison with the non-supplemented piglets.

## 5. Conclusions

Our results provide the first clues on the medium-term effect on immune response of a mild LPS challenge in piglets fed a control diet or control diet supplemented with a mixture of functional amino acids. It was found that stress biomarkers of inflammation and hormonal status, such as haptoglobin and cortisol, are exacerbated by the LPS challenge and can be partially reversed by the addition of an amino acid mixture. In contrast, TNF-α was not affected by experimental treatments and the proteomics approach did not reveal important alterations of the protein profile in response to the challenge or supplementation. However, this latter analysis still suggests a downregulation of the inflammation pathway in piglets receiving the amino acid mixture. Taken together, these data indicate that piglets fed diets supplemented with this amino acid mixture are less susceptible to the negative effects of LPS challenge. However, the number of piglets included in this study was rather small and, therefore, larger-scale studies are needed to investigate if the modulation in inflammation markers can translate into significant effects on animal performance.

## Figures and Tables

**Table 1:** Ingredients (g·kg^−1^) and proximate chemical composition of diets used in the trial.

Ingredients	CTRL and LPS	LPS+MIX
Wheat	200.0	200.0
Corn	381.6	381.6
Soybean meal (48)	268.0	268.0
Sweet dry whey	70.0	70.0
Soybean oil	30.0	30.0
L-Lys	6.4	6.4
DL-Met	2.7	2.7
L-Thr	2.8	2.8
L-Trp	1.1	1.1
L-Val	3.2	3.2
CaCO_3_	10.0	10.0
Dicalcium Phosphate	16.0	16.0
Sodium bicarbonate	2.2	2.2
NaCl	3.0	3.0
Vitamin trace mineral mix ^(1)^	3.0	3.0
L-Arg	0.0	1.25
L-Leu	0.0	0.50
L-Val	0.0	0.25
L-Ile	0.0	0.25
L-Cys2	0.0	0.75
SID * LYS pig	13.50	13.50
SID THR pig	8.78	8.78
SID MET pig	5.28	5.28
SID CYS pig	2.83	3.58
SID TRP pig	2.97	2.97
SID ILE pig	7.16	7.41
SID VAL pig	10.80	11.05
SID LEU pig	13.54	14.04
SID ARG pig	10.90	12.15
**Chemical composition**		
Dry matter (%)	89.7	89.2
Ash (%)	5.56	5.71
Crude protein (%)	19.75	20.05
Ether Extract (%)	5.92	5.80
NE (MJ/kg)	10.53	10.53

^(1)^ Vitamin and trace mineral supplied per kilogram of diet: Vit. A, 2500 IU; Vit. D3, 200 IU; Vit. E, 20 IU; Vit. C, 200 mg; Vit. B1, 1.5 mg; Vit. B2, 5 mg; Vit. B3, 30 mg; Vit. B5, 15 mg; Vit. B6, 2.5 mg; Vit. B9, 0.5 mg; Vit. B12, 0.03 mg; Vit. K3, 1 mg; Biotin, 80 mg; choline (chloride): 300 mg; I, 1 mg as potassium iodate; Mn, 50 mg as manganese (oxide); Fe, 120 mg as ferrous carbonate; Zn, 140 mg as zinc (oxide); Cu, 160 mg as copper sulphate; Se, 0.3 mg as sodium selenite; Co, 0.5 mg as cobalt carbonate. Groups are: CTRL, the control group; LPS, the LPS-challenged diet group; LPS+MIX, the LPS-challenged diet supplemented with arginine, BCAA and cystine mixture group. * SID: standardised ileal digestibility.

**Table 2:** Weekly performance of the piglets included in the study.

	CTRL	LPS	LPS+MIX	SEM	*p*-Value
Week before LPS challenge (day 0–7)					
Initial weight (kg)	8.46	8.39	7.95	0.19	0.498
Final weight (kg)	9.98	9.71	9.88	0.30	0.945
Average daily gain (g/d)	217.5	188.8	275.3	25.7	0.385
Feed intake (g/d)	366.2	333.3	301	30.7	0.694
Week after LPS challenge (day 7–14)					
Final weight (kg)	13.05	11.85	12.11	0.42	0.512
Average daily gain (g/day)	438.9	306.1	318.8	32.0	0.184
Feed intake (g/day)	484.2	581.7	453.2	42.0	0.540
Last three weeks of the study (day 14–35)					
Final weight (kg)	27.07	25.79	27.08	0.75	0.772
Average daily gain (g/day)	667.5	663.9	712.8	20.3	0.542
Feed intake (g/ day)	1054.6	1149.2	1110.9	48.3	0.686

Groups are: CTRL, the control group; LPS, the LPS-challenged group; LPS+MIX, the LPS-challenged supplemented with arginine, branched-chain amino acids (BCAA) and cystine mixture group. SEM: standard error of the mean

**Table 3:** Effect of treatments on inflammatory markers and hormonal status.

	CTRL	LPS	LPS+MIX	SEM	*p*-Value
Inflammatory markers					
Day 10					
TNF-α (pg/mL)	129.9	134.8	142.4	10.08	0.886
Haptoglobin (mg/dL)	23.16 ^a^	31.91 ^b^	32.08 ^b^	0.996	<0.001
Day 35					
TNF-α (pg/mL)	61.38	78.60	69.63	4.996	0.446
Haptoglobin (mg/dL)	20.64 ^a^	19.84 ^ab^	13.59 ^b^	1.317	<0.05
Hormones					
Day 10					
Cortisol (µg/dL)	2.700 ^a^	3.463 ^c^	1.711 ^b^	0.158	<0.001
IGF-1 (µg/L)	59.07 ^a^	92.51 ^b^	110.6 ^c^	4.489	<0.001
Day 35					
Cortisol (µg/dL)	3.167	3.243	2.690	0.1922	0.447
IGF-1 (µg/L)	159.8 ^a^	143.8 ^a^	231.3 ^b^	9.708	<0.001

Groups are: CTRL, the control group; LPS, the LPS-challenged group; LPS+MIX, the LPS-challenged supplemented with arginine, branched-chain amino acids (BCAA) and cystine mixture group. ^a,b,c^ Mean values within a row with different letters are significantly different *(p* < 0.05). SEM: standard error of the mean, TNF-α: tumor necrosis factor-α, IGF-1: insulin-like growth factor-1.

**Table 4:** Differential protein abundance in serum on day 35 between CTRL and LPS groups, and LPS and LPS+MIX groups.

Accession Number	Protein Name	Peptide Count	Unique Peptides	ANOVA	Fold Change (LPS/CTRL)
				*p*-Value	
**CTRL vs. LPS**					
A0A5G2QEV5	Inter-alpha-trypsin inhibitor heavy chain H2	21	21	0.03	0.876
F1SL22	von Willebrand factor	9	9	0.04	0.4
A0A4x1U519	C1q domain-containing protein	2	2	0.04	0.59
A0A287AAW7	C1q domain-containing protein	1	1	0.02	0.642
**LPS vs. LPS+MIX**					
A0A287A1M4	Uncharacterised protein	11	7	0.04	0.734
A0SEH1	Complement component	9	1	0.05	1.686
A0A5G2QLJ8	Complement C1q B chain	2	2	0.03	0.704
A0A4x1VG41	Beta-2-microglobulin	1	1	0.03	0.01
A0A287A359	Uncharacterised protein	1	1	0.03	0.814
B2CNZ7	Cathepsin B	1	1	0.04	0.716
A0A286ZKE0	Uncharacterised protein	1	1	0.03	0.867

Groups are: CTRL, the control group; LPS, the LPS-challenged group; LPS+MIX, the LPS-challenged supplemented with arginine, branched-chain amino acids (BCAA) and cystine mixture group.

## Data Availability

The data presented in this study are not publicly available due to privacy restrictions.

## Supplementary Materials

The following are available online at https://www.mdpi.com/article/10.3390/ani11041143/s1, Table S1: full proteomics results
